# The Impact of Comorbidities on Health-Related Quality of Life Among Patients with Rheumatoid Arthritis

**DOI:** 10.3390/healthcare14020256

**Published:** 2026-01-20

**Authors:** Adriana Liliana Vlad, Corina Risca Popazu, Alina-Maria Lescai, Daniela-Ioanina Prisacaru, Doina Carina Voinescu, Alexia Anastasia Stefania Baltă

**Affiliations:** 1 Faculty of Medicine and Pharmacy, Research Centre in the Medical-Pharmaceutical Field, “Dunărea de Jos” University of Galați, Strada Domnească 47, 800008 Galați, Romania; adriana.vlad.mg3.4@gmail.com (A.L.V.); corinapopazu@yahoo.com (C.R.P.); carinavoinescu@gmail.com (D.C.V.); alexiabalta@yahoo.ro (A.A.S.B.); 2 “St. Apostle Andrei” County Emergency Clinical Hospital, 800578 Galați, Romania; 3 Tübinger Akademie für Verhaltenstherapie, David-von-Stein-Weg 26, 72072 Tübingen, Germany; ioaninaprisacaru@gmail.com

**Keywords:** rheumatoid arthritis, comorbidities, quality of life, integrated care, chronic autoimmune disease

## Abstract

**Background.** Rheumatoid arthritis (RA) is a chronic autoimmune disease frequently accompanied by cardiovascular, respiratory, skeletal, psychiatric, and neoplastic comorbidities that are associated with higher morbidity and poorer health-related quality of life (HRQoL). This study evaluated the associations between comorbidities and patient-reported physical health, emotional distress, daily functioning, and social relationships in adults with RA and explored patient-reported unmet needs relevant to integrated care. **Methods.** We conducted a cross-sectional survey among 286 adults with physician-confirmed RA, using a structured questionnaire (ICRA-Q) administered between June and July 2025 via online platforms and in-hospital supervised completion. The survey captured demographics, patient-reported physician-diagnosed comorbidities (current and/or past), perceived disease impact, functional limitations, emotional and social consequences, access to treatment, financial burden, and support needs. Analyses included descriptive statistics, χ^2^ tests, *t*-tests/ANOVA, effect sizes (Cramer’s V and standardized mean differences), and multivariable logistic regression to explore predictors of high HRQoL impact and high difficulty in disease management. An exploratory classification into high-risk phenotypes was performed using predefined clinical, psychological, and socioeconomic criteria. **Results.** Most participants (98.6%) reported at least one comorbidity, most commonly hypertension, osteoporosis, and cardiovascular disease. Higher comorbidity burden and depression/anxiety were strongly associated with higher pain, reduced mobility, emotional distress, and financial strain. Exploratory high-risk phenotypes (severe somatic multimorbidity, high psychological vulnerability, high socioeconomic burden, and a composite very high-risk profile) were associated with poorer HRQoL indicators. Younger age, shorter disease duration, and higher perceived social support were associated with lower perceived burden. **Conclusions.** In this cross-sectional, patient-reported study, comorbidity burden—particularly psychological comorbidity—was strongly associated with poorer HRQoL and greater management difficulty in RA. These findings support the need for multidisciplinary, integrated care pathways; however, subgroup phenotypes should be considered exploratory and require external validation.

## 1. Introduction

Rheumatoid arthritis (RA) is a chronic systemic autoimmune disease characterized by persistent synovial inflammation, progressive joint destruction, and extra-articular manifestations [1]. The disease has a global prevalence of approximately 0.5–1% and is associated with a significantly reduced life expectancy compared with the general population [2,3]. Although the introduction of disease-modifying antirheumatic drugs (DMARDs) and, more recently, biologic and targeted synthetic DMARDs, has revolutionized disease control and reduced disability rates, RA patients still exhibit higher morbidity and mortality than individuals without RA [4].

The excess mortality in RA is largely attributable to the high prevalence of comorbidities that accompany the disease [5]. Epidemiological studies consistently demonstrate that more than half of RA patients develop at least one clinically relevant comorbidity during their lifetime [6,7]. Among these, cardiovascular diseases (CVDs) remain the most frequent and clinically significant [6]. RA patients face a 1.5-2-fold higher risk of myocardial infarction and cerebrovascular events compared to the general population [8]. This increased cardiovascular burden persists even after adjusting for traditional risk factors, which highlights the pathogenic role of systemic inflammation, endothelial dysfunction, and pro-atherogenic lipid alterations [9]. Moreover, long-term corticosteroid therapy contributes to metabolic dysregulation and accelerates vascular injury, further amplifying cardiovascular risk [10]. Consequently, cardiovascular disease is recognized as the leading cause of death in RA cohorts [11].

Respiratory complications also account for a significant proportion of RA-related mortality [12]. Interstitial lung disease (ILD), in particular, represents one of the most severe extra-articular manifestations of RA, frequently associated with a poor prognosis [13]. RA patients are also at higher risk for chronic obstructive pulmonary disease (COPD) and recurrent respiratory infections, both of which further increase hospitalization rates and mortality [14].

Skeletal comorbidities are another important contributor to disability in RA [15]. Osteoporosis is highly prevalent due to chronic inflammation, glucocorticoid therapy, and reduced mobility, and is a major risk factor for fragility fractures [16]. These fractures significantly impair quality of life and lead to higher mortality [17].

In addition to physical comorbidities, psychiatric disorders such as depression and anxiety are observed in up to 40% of RA patients [18]. Depression is strongly associated with higher disease activity, worse functional outcomes, and poor adherence to treatment, thereby indirectly increasing morbidity and mortality [19].

Neoplastic conditions are also reported at higher rates in RA patients, particularly lymphoproliferative malignancies and non-melanoma skin cancers [20]. The pathogenesis appears to involve a combination of chronic immune activation and the potential carcinogenic effect of prolonged immunosuppressive therapy [21].

The cumulative impact of comorbidities in RA is profound. Large registry-based studies have confirmed that comorbidity burden is among the strongest predictors of both mortality and functional disability [22]. For example, data from Scandinavian registries demonstrated that RA patients have standardized mortality ratios up to 50% higher than the general population, mainly due to cardiovascular and respiratory causes [23]. Similarly, Australian cohort studies identified pneumonia and interstitial lung disease as the leading causes of death in long-term follow-up of RA patients [24]. Even in the biologic era, mortality rates remain elevated, which indicates that inflammation control alone is insufficient to normalize survival [25].

Moreover, comorbidities significantly affect treatment outcomes. Patients with multiple concomitant conditions are less likely to achieve remission, are at increased risk of treatment discontinuation, and experience higher rates of adverse events during biologic or targeted therapy [26]. As a result, the management of RA increasingly requires an integrated, multidisciplinary approach [27]. Current international recommendations emphasize the importance of systematic cardiovascular risk screening, early detection of pulmonary involvement, bone health monitoring, psychosocial support, and cancer surveillance [28].

The primary aim of the study is to evaluate the impact of comorbidities on patients with rheumatoid arthritis, focusing on how these associated conditions influence physical health, psychological well-being, daily activities, social relationships, and overall quality of life. This objective is pursued through a structured questionnaire designed to capture patients’ perspectives regarding the prevalence and type of comorbidities, the degree to which these conditions complicate disease management, and the challenges faced in accessing healthcare services and treatments. The secondary aim is to identify unmet needs and potential strategies to improve integrated care, including medical monitoring, access to effective therapies, patient education, psychological support, and financial assistance programs, with the ultimate goal of optimizing patient outcomes and quality of life in rheumatoid arthritis.

## 2. Materials and Methods

In this manuscript, the term *health-related quality of life (HRQoL)* is used consistently to refer to patients’ perceived physical, psychological, and social functioning as influenced by rheumatoid arthritis and associated comorbidities. The broader term *quality of life* is used only when referring to general concepts or patient-reported perceptions, and not interchangeably with HRQoL. Similarly, *psychological distress* is used as an umbrella term encompassing symptoms of depression, anxiety, and emotional burden. The term *emotional impact* is employed to describe patients’ subjective emotional responses, while *psychological status* refers specifically to mental health–related aspects affecting disease management.

### 2.1. Design

This study was conducted as a cross-sectional, non-interventional survey designed to evaluate the prevalence of comorbidities and their impact on health status, disease management, and health-related quality of life (HRQoL) among patients with rheumatoid arthritis. Data were collected using a structured, self-administered questionnaire distributed between June and July 2025, after obtaining ethical approvals from the participating institutions, via Google Forms. The survey link was disseminated through social media platforms and patient communication channels, including WhatsApp support groups and email. In addition, a subset of questionnaires was completed by hospitalized patients under the supervision of medical staff, to facilitate participation among patients with limited digital access. The study design was chosen to capture a wide range of patient-reported outcomes without interfering with ongoing medical management; however, the mixed recruitment strategy may introduce selection bias, which is addressed in the Limitations section.

### 2.2. Participants

All adult patients (≥18 years) with a physician-confirmed diagnosis of rheumatoid arthritis were eligible to participate. A total of 286 complete questionnaires were returned and included in the final analysis. Exclusion criteria were lack of a confirmed RA diagnosis, incomplete questionnaire submission, or refusal to provide informed consent.

All participants provided informed consent prior to inclusion in the study. For participants recruited online, informed consent was obtained electronically through a mandatory consent form integrated at the beginning of the online questionnaire. Participants were required to actively confirm their agreement before accessing and completing the survey.

For hospitalized participants, written informed consent was obtained in person under the supervision of medical staff prior to questionnaire administration.

Participation was voluntary, and patients were informed that they could withdraw from the study at any time without any consequences. Data confidentiality was strictly maintained, with all responses anonymized and processed in compliance with the General Data Protection Regulation (GDPR).

Recruitment was consecutive, and all patients who accessed the survey during the predefined study period were included. Participation was voluntary and based on self-selection, which may have influenced sample characteristics.

Participants were stratified by demographic characteristics (age, sex, and living environment) and by disease duration to allow comparative analyses of comorbidity burden across clinical and sociodemographic subgroups.

### 2.3. Data Collection

Data collection was carried out between June and July 2025, following institutional ethics approval. Participants independently completed the structured questionnaire, which required approximately 10 min.

For online participants, informed consent was obtained electronically before accessing the questionnaire. For hospitalized participants, consent was obtained prior to supervised questionnaire completion.

### 2.4. Instrument

Impact of Comorbidities in Rheumatoid Arthritis Questionnaire (ICRA-Q)—a structured, self-administered questionnaire specifically developed for this study to evaluate the prevalence and impact of comorbidities among patients with rheumatoid arthritis (RA). The instrument was designed to capture patient-reported outcomes across multiple domains, including physical health, psychological well-being, social relationships, access to healthcare, and financial burden (see Appendix A).

The questionnaire consisted of 29 items grouped into ten sections:

(1) demographic data (age, gender, living environment, disease duration);
(2) self-reported physician-diagnosed comorbidities (current and/or past), including hypertension, cardiovascular disease, diabetes, osteoporosis, obesity, depression/anxiety, and others;
(3) impact of comorbidities on health status;
(4) perceived difficulty in managing RA with comorbidities;
(5) treatment and access to healthcare;
(6) psychological and emotional impact;
(7) social and occupational impact;
(8) access to medical information and education;
(9) financial burden of disease management;
(10) perceived support needs.

Responses included both single-choice and multiple-choice formats, as well as Likert-type scales (ranging from “not at all” to “very much”) to assess the severity of the impact in different domains. Open-ended items were also included, allowing patients to provide individual comments or suggestions.

The Impact of Comorbidities in Rheumatoid Arthritis Questionnaire (ICRA-Q) was specifically developed for this study as an exploratory, disease-focused instrument intended to capture the multidimensional patient-perceived burden of comorbidities. While pilot testing demonstrated good internal consistency (Cronbach’s alpha = 0.89), the questionnaire has not undergone full psychometric validation, such as confirmatory factor analysis, test–retest reliability assessment, or evaluation of convergent and divergent validity in relation to established instruments.

Accordingly, the ICRA-Q should be considered an exploratory tool, and results derived from this instrument are interpreted as hypothesis-generating rather than confirmatory. The questionnaire was designed to assess domains that are not comprehensively covered by existing validated instruments, including perceived difficulty in managing multiple conditions, unmet integrated care needs, and the socioeconomic and financial burden associated with multimorbidity.

Validated generic and disease-specific instruments (e.g., HAQ, SF-36, EQ-5D, WHOQOL-BREF) were not used in this study because they primarily assess functional status or general health-related quality of life and do not specifically address the perceived impact of comorbidity burden, care integration challenges, or financial strain. Future research should incorporate validated HRQoL instruments alongside further psychometric validation of the ICRA-Q.

Unlike standardized generic tools such as WHOQOL-BREF or MFIS, the ICRA-Q was designed to be disease-specific, focusing on the multidimensional burden of comorbidities in RA patients. Generic HRQoL or functional tools (e.g., EQ-5D, SF-36, HAQ) were not used because they do not capture perceived multimorbidity management difficulty, unmet integrated care needs, or disease-related financial burden within a single framework. Future studies should incorporate validated instruments alongside further psychometric evaluation of the ICRA-Q.

Comorbidities were assessed using self-reported information provided by participants. Respondents were asked to indicate comorbid conditions that had been diagnosed by a physician at any time (current or past), based on their medical history. No independent clinical verification through medical records, laboratory data, or imaging was performed. Therefore, comorbidity data reflect patient-reported physician diagnoses rather than objectively confirmed current disease status.

As a result, the reported prevalence captures lifetime exposure to comorbid conditions rather than point prevalence of active comorbidity.

### 2.5. Statistics

Data were analyzed using IBM SPSS Statistics for Windows, Version 26.0 (IBM Corp., Armonk, NY, USA). Descriptive statistics (absolute frequencies, percentages, means, and standard deviations) were used to summarize demographic characteristics, comorbidity prevalence, and patient-reported outcomes.

Inferential statistical methods included:

- χ^2^ tests to evaluate associations between categorical variables (e.g., comorbidity presence and perceived quality of life).
- *t*-tests or ANOVA for comparisons of continuous variables across subgroups (e.g., age groups, disease duration).
- Logistic regression to identify predictors of poor health status or high comorbidity burden.
- Effect size calculations using Cramer’s V for categorical associations and standardized mean differences for continuous variables to quantify the strength of comorbidity impact.
- Exploratory subgroup classification into high-risk phenotypes based on predefined clinical, psychological, and socioeconomic criteria.

A *p*-value of <0.05 was considered statistically significant. Missing data were excluded case-wise. Given the very small number of participants without comorbidities, direct comparisons with this group were interpreted cautiously, and greater emphasis was placed on analyses based on comorbidity burden and multivariable models with confidence intervals.

Given the very small number of participants without comorbidities (n = 4), this group was not considered a robust control group. Comparisons involving this subgroup were therefore interpreted with caution. Greater emphasis was placed on analyses comparing different levels of comorbidity burden (0–1, 2–3, ≥3 comorbidities) and on multivariable logistic regression models with 95% confidence intervals, which provide more stable and informative estimates.

Exploratory subgroup classification (high-risk profiles). To explore patterns of multidimensional vulnerability, we conducted an exploratory subgroup classification based on clinically plausible and patient-reported domains: comorbidity burden, psychological vulnerability, and socioeconomic strain. The criteria were specified a priori for the purpose of within-sample descriptive profiling and were not derived by data-driven clustering algorithms. Specifically, we defined: (1) severe somatic multimorbidity (≥3 comorbidities including ≥1 major cardiometabolic condition), (2) high psychological vulnerability (self-reported depression/anxiety and high emotional impact/psychological influence on disease management), (3) high socioeconomic burden (high/very high perceived financial burden plus frequent financial difficulties and frequent work limitations), and (4) a composite very high-risk profile (≥3 comorbidities plus depression/anxiety plus high financial burden). This analysis is hypothesis-generating and has not been externally validated; therefore, these profiles should not be interpreted as clinically actionable phenotypes.

### 2.6. Ethics

The study was conducted in accordance with the ethical principles of the Declaration of Helsinki (1964, revised), the Belmont Report (1979), and the ICH–Good Clinical Practice (GCP) guidelines. The research protocol was reviewed and approved by the Ethics Committee of the “Sf. Apostol Andrei” Clinical Emergency County Hospital, Galați (approval No. 2008/03.02.2025) and by the Ethics Committee of the Faculty of Medicine and Pharmacy, “Dunărea de Jos” University of Galați (approval obtained in May 2025). All participants provided informed consent before completing the questionnaire. Participation was voluntary, and patients were informed that they could withdraw at any time without any consequences. Data confidentiality was strictly maintained, with all responses anonymized and processed in compliance with the General Data Protection Regulation (GDPR).

## 3. Results

### 3.1. Sociodemographic Characteristics

A total of 286 patients with rheumatoid arthritis (RA) completed the questionnaire. The majority of respondents were aged between 41 and 60 years, with 40.6% in the 41–50 age group and 38.1% in the 51–60 age group, while only 14.7% were younger than 40 years and 6.6% were recently diagnosed patients under 30 years old. Participants over 60 years represented 38.1% of the total sample, reflecting the chronic and long-term burden of the disease in older age groups.

Gender distribution was strongly imbalanced, with a predominance of females (85.0%) compared to males (15.0%), which is consistent with the known higher prevalence of RA in women. Most patients lived in urban areas (87.8%), while only 12.2% reported residence in rural regions.

Regarding disease duration, one-third of participants (33.9%) had been diagnosed within the last 1–5 years, while almost half of the patients (46.5%) reported living with RA for 6–10 years. A smaller subgroup of 12.9% had been diagnosed less than one year prior to the survey, and 12.9% reported a disease duration of more than 10 years.

These findings highlight a patient population composed predominantly of middle-aged and older women, residing mainly in urban areas, and living with RA for an extended period, which increases the likelihood of accumulating comorbidities and experiencing disease-related complications (Table 1).

### 3.2. Comorbidities and Their Impact

Almost all participants (98.6%) reported at least one comorbidity in addition to rheumatoid arthritis, while only 1.4% declared no other associated conditions. The most frequently diagnosed comorbidities were hypertension (70.9%), osteoporosis (60.0%), and cardiovascular disease (58.2%), followed by obesity (40.0%), diabetes mellitus (29.5%), and depression or anxiety (22.1%). These findings reflect the strong overlap between RA and major chronic conditions that further impair patients’ health status and quality of life.

Regarding the perceived burden of comorbidities, the majority of respondents (59.6%) reported that 2–3 associated conditions significantly affected their quality of life, while 20.4% reported only one comorbidity and 18.2% declared none. A smaller but relevant subgroup (20.4%) experienced the cumulative impact of more than three comorbidities.

Most patients perceived their comorbidities as having a high negative impact on their general health, with 67.8% rating the influence as “much” and 22% as “very much.” Only 4.2% of patients reported that comorbidities affected them little or not at all. Similarly, disease management was considered more difficult in the presence of comorbidities, with 68.2% describing it as “difficult” and 28.3% as “very difficult.”

The main difficulties attributed to comorbidities included increased pain (98.3%), reduced mobility (95.5%), fatigue (75.9%), emotional problems such as anxiety and depression (40.9%), and financial burden due to treatment costs (23.1%). Nearly all respondents (97.9%) confirmed that they were under treatment for their comorbidities in addition to RA therapy.

When asked about healthcare access, most patients evaluated their situation as good (92.3%), with only a minority considering it merely satisfactory. The perceived medical support was also high, with 78.7% reporting that they received sufficient help from their physicians. Nevertheless, patients expressed unmet needs, particularly for more effective treatments (96.2%), better medical monitoring (93.7%), and improved education about managing multiple conditions (79.7%). Emotional and psychological support was also highlighted by 58.4% of participants (Table 2).

### 3.3. Psychological and Social Impact

Table 3 presents the psychological and social impact of comorbidities among the study participants. The majority of patients reported that comorbidities affected their emotional state considerably: 65.4% stated “much” and 27.3% “moderately”, while only a very small fraction (2.8%) indicated little or no influence.

Symptoms of depression or anxiety were highly prevalent, with most respondents (80.4%) experiencing them occasionally, while 8.7% reported frequent symptoms. Only 10.8% declared rare episodes, and none denied their occurrence. Despite this high prevalence, only a minority sought psychological support, with 6.3% using counselling services frequently, while the majority either did not seek help or expressed a desire for such support in the future. Participation in support groups was even lower, suggesting unmet needs for structured psychosocial interventions.

The psychological condition of patients was closely linked with disease management: 75.5% acknowledged that their emotional state influenced RA management to a significant degree, while only 0.4% stated no effect at all.

From a social perspective, the burden of comorbidities was evident. Most patients reported that their relationships with family, friends, or colleagues were negatively affected, with 73.1% rating the impact as “much” and 19.9% as “very much.” Occupational functioning was also impaired: 84.6% of patients reported occasional difficulties maintaining a job or daily activities, and 7.0% experienced such difficulties frequently.

Social understanding was relatively high: 88.8% of participants felt that people around them partially understood their difficulties, while only 9.4% considered themselves fully understood. However, a small group (1.8%) reported that those around them did not understand their health challenges at all.

### 3.4. Access to Medical Information, Education and Financial Burden

Table 4 illustrates patients’ access to medical information, educational resources, and their perception of the financial burden related to the management of rheumatoid arthritis (RA) and associated comorbidities.

Most respondents reported a good understanding of the impact of comorbidities on RA, with 89.2% stating they understood it well or very well, while only a small minority (6.3%) reported limited understanding. Regarding sources of information, the rheumatologist (99%) and the general practitioner (93.4%) were the most frequently consulted, followed by the internet (45.8%), patient associations (11.5%), and family or friends (9.8%).

When asked about willingness to participate in educational programs on RA and comorbidities, the majority expressed interest, with 46.9% answering “maybe, depending on the schedule”, 36.7% agreeing if free of charge, and 16.1% stating they would definitely participate. Only one respondent declared disinterest. These findings suggest a strong unmet need for structured patient education programs.

The financial burden was also significant. Most participants considered the management of RA and comorbidities as high (69.6%) or very high (23.4%), while only a few reported moderate (5.9%) or low (1.1%) costs. The main reported expenses were for medications (96.2%), diagnostic tests and imaging (86.4%), medical consultations (85.3%), and physical therapy/rehabilitation (46.9%).

Despite the heavy economic load, most patients managed to cover costs only with difficulty. While 79.7% reported occasional financial difficulties, 10.5% experienced frequent challenges, and only 2 patients (0.7%) stated they had no difficulties at all.

Overall, these results demonstrate that RA patients not only face a substantial medical and psychosocial burden but also encounter considerable financial strain and express a strong demand for improved education, guidance, and support programs.

### 3.5. Support Needs

Table 5 summarizes patients’ perspectives regarding the types of support considered most useful for improving quality of life in the context of rheumatoid arthritis and its comorbidities. The majority of participants highlighted the importance of better access to effective and financially affordable treatments (96.2%) and more frequent consultations with medical specialists (95.5%). These findings indicate a strong patient demand for continuous medical monitoring and improved healthcare coverage.

Equally important, a large proportion of patients expressed the need for structured rehabilitation programs (88.8%), reflecting the significant physical limitations associated with RA and comorbid conditions. Additionally, psychological support or peer support groups (68.2%) were recognized as valuable for coping with the emotional burden of the disease. Finally, more than half of respondents (53.5%) considered expanded medical education programs essential for better disease self-management, underlining the need for comprehensive, patient-centred educational strategies.

Overall, these findings suggest that RA patients perceive their quality of life as being closely linked not only to access to treatment but also to multidimensional support systems that integrate physical, psychological, and educational interventions.

The chi-square analysis revealed a statistically significant association between the number of comorbidities and patients’ perceived impact on quality of life (χ^2^(3) = 36.41, *p* < 0.001). Patients with two or more comorbidities were substantially more likely to report a high or very high impact on their quality of life compared to those with none or only one comorbidity. Specifically, more than 80% of patients with two to three comorbidities and 91.4% of those with more than three comorbidities perceived a severe negative effect, whereas this proportion decreased to 53.8% in patients with one comorbidity and to only 20% in those without any comorbidities. These findings emphasize that the cumulative burden of comorbidities strongly correlates with diminished quality of life in rheumatoid arthritis patients (Table 6).

The independent samples t-test demonstrated a statistically significant difference in perceived quality of life between patients with comorbidities and those without (t(284) = 5.37, *p* < 0.001). However, the group without comorbidities comprised only four participants, which limits the statistical reliability and generalizability of this comparison. Accordingly, this result should be interpreted as descriptive rather than confirmatory, and greater interpretative weight was given to analyses examining comorbidity burden across multiple categories and to regression models reporting confidence intervals (Table 7).

An important limitation of the comparative analyses relates to the very small number of participants without comorbidities (n = 4), which constrains the statistical reliability of using this subgroup as a reference category. Although the independent samples t-test indicated a statistically significant difference in perceived quality of life between patients with and without comorbidities, estimates derived from such a small comparator group are inherently unstable and should not be overinterpreted or generalized.

Accordingly, conclusions regarding the impact of the presence versus absence of comorbidities were interpreted cautiously and considered descriptive rather than confirmatory. Greater interpretative weight was therefore placed on analyses examining the number and type of comorbidities, including the ANOVA models assessing comorbidity burden across multiple categories and the multivariable logistic regression analyses, which provide more robust and stable estimates through the use of larger subgroup sizes and confidence intervals.

These analyses consistently demonstrated that increasing comorbidity burden and specific high-impact comorbidities were associated with poorer quality-of-life outcomes and greater difficulty in disease management, independent of the very small “no comorbidity” subgroup. Future studies should aim to include larger and more balanced comparator groups to strengthen inference regarding differences between patients with and without comorbidities.

The one-way ANOVA revealed a significant effect of age on perceived general health impact among patients with rheumatoid arthritis. Participants aged under 40 reported the lowest mean scores (M = 3.45, SD = 0.68), indicating a comparatively lower perceived health burden. The mean score increased progressively across the 41–50 (M = 3.78, SD = 0.61) and 51–60 (M = 4.12, SD = 0.55) groups, reaching the highest value in the over-60 group (M = 4.20, SD = 0.50). Post hoc analyses confirmed that patients in the 51–60 and >60 age groups experienced a significantly greater negative health impact compared with younger participants. These results suggest that advancing age is strongly associated with a worsening perception of general health in RA patients, reflecting the cumulative burden of disease and age-related comorbidities (Table 8).

ANOVA: F(3, 282) = 18.27, *p* < 0.001, partial η^2^ = 0.163

Post hoc (Tukey):

- >60 > <40 (*p* < 0.001), >60 > 41–50 (*p* = 0.002)
- 51–60 > <40 (*p* < 0.001), 51–60 > 41–50 (*p* = 0.011)
- 41–50 vs. <40 (*p* = 0.094)

*Note:* The <30 group (n = 1) was merged with 30–40 to satisfy ANOVA assumptions.

The one-way ANOVA demonstrated a significant effect of disease duration on perceived general health impact. Patients with a disease history of less than one year reported the lowest mean score (M = 3.50, SD = 0.65), indicating a comparatively lower perceived burden. The mean score increased steadily with longer disease duration, reaching 3.79 (SD = 0.62) in those with 1–5 years of RA, 4.08 (SD = 0.57) in the 6–10 years group, and peaking at 4.22 (SD = 0.52) in patients with more than 10 years of disease. Post hoc comparisons confirmed that patients with over 10 years of RA perceived their health as significantly worse than those with shorter disease duration, particularly those newly diagnosed. These findings suggest that the cumulative burden of RA and associated comorbidities intensifies over time, leading to a progressive deterioration in perceived health status (Table 9).

ANOVA: F(3, 282) = 14.91, *p* < 0.001, partial η^2^ = 0.137

Post-hoc (Tukey):

- >10 years > <1 year (*p* < 0.001) and >1–5 years (*p* = 0.004)
- 6–10 years > <1 year (*p* = 0.003)
- 6–10 years vs. 1–5 years (*p* = 0.078)
- >10 years vs. 6–10 years (*p* = 0.41)

The one-way ANOVA showed a significant effect of age on perceived general health impact, with older participants reporting worse scores than younger counterparts (F(3, 282) = 18.27, *p* < 0.001; partial η^2^ = 0.163). Post hoc comparisons indicated that both the 51–60 and >60 groups had significantly higher (worse) impact scores than the <40 and 41–50 groups, suggesting that age is associated with a progressively greater perceived health burden.

Similarly, disease duration exerted a significant effect (F(3, 282) = 14.91, *p* < 0.001; partial η^2^ = 0.137). Patients with >10 years of RA reported markedly worse impact than those with shorter disease histories, and those with 6–10 years reported worse outcomes than newly diagnosed patients. These findings are consistent with a cumulative burden model, whereby longer exposure to RA and its comorbidities corresponds to a greater perceived deterioration in general health.

### 3.6. High-Risk Patient Subgroups

To identify specific patterns among patients experiencing a substantial multidimensional impact from comorbidities, participants were classified into distinct clinical subgroups based on the combination of comorbidity burden, psychological vulnerability and socioeconomic strain. This analytical approach allowed the identification of several high-risk phenotypes characterized by pronounced impairment in quality of life, difficulty in disease management and significant functional limitations. Table 10 presents the characteristics of these subgroups and the key differences identified between them.

Table 10 highlights the presence of clearly defined clinical subgroups with differing levels of vulnerability. The subgroup characterized by severe somatic multimorbidity shows considerable deterioration in quality of life, primarily due to the presence of major cardiovascular and metabolic comorbidities that substantially influence the course of rheumatoid arthritis.

Patients classified within the subgroup of high psychological vulnerability demonstrate that emotional distress contributes independently to functional impairment, even in individuals with fewer somatic comorbidities. This finding indicates a clear need for structured psychological interventions and professional support.

The subgroup experiencing high socioeconomic burden presents the greatest proportion of difficulties in work related tasks and daily activities. This suggests that financial barriers amplify the impact of comorbid conditions, potentially affecting treatment adherence and long term disease management.

The very high-risk composite phenotype combines severe somatic comorbidity, psychological distress and financial strain. This group demonstrates the poorest outcomes across all evaluated domains including physical health, emotional wellbeing, social functioning and economic stability. These patients represent a priority group for targeted, integrated and multidisciplinary interventions.

### 3.7. Identified Protective Factors

Although much of the existing literature focuses on risk factors that worsen outcomes in rheumatoid arthritis, it is equally important to identify characteristics that may buffer the negative effects of comorbidities. Several protective factors emerged in this study, including demographic and psychosocial variables associated with better perceived health, improved emotional resilience and reduced disease burden. These protective elements provide insight into potential targets for supportive interventions. Table 11 presents the principal protective factors observed in the study population and their associations with reduced impact on quality of life.

The results indicate that several protective factors mitigate the perceived burden of rheumatoid arthritis in the presence of comorbidities. Younger age and shorter disease duration were associated with lower levels of functional and psychological impairment, which is consistent with the notion that early disease stages are more manageable and have a lower cumulative burden. The absence of psychological distress emerged as one of the strongest protective factors, reinforcing the central role of mental health in determining patient outcomes.

Patients with no more than one comorbidity reported substantially better quality of life compared with those with multiple associated conditions, highlighting the importance of early identification and prevention of additional comorbidities. High perceived social support was also linked to reduced emotional strain, suggesting that interpersonal understanding and family involvement may serve as important buffers against disease-related stress. Finally, good access to healthcare services was associated with improved disease management and lower perceived difficulty in coping with comorbidities.

Together, these findings suggest that protective factors operate at demographic, clinical, psychological and social levels and may provide valuable targets for interventions aimed at enhancing resilience and improving long-term outcomes in patients with rheumatoid arthritis.

### 3.8. Comorbidities with the Strongest Impact, Ordered by Effect Size

To determine which comorbidities contribute most significantly to the perceived health burden in patients with rheumatoid arthritis, the main associated conditions were ranked according to their effect size. This approach provides a clearer understanding of the relative influence of each comorbidity on quality of life and functional outcomes. Effect sizes were computed using Cramer’s V for categorical associations and standardized mean differences where appropriate. Table 12 presents the comorbidities in descending order of their impact on quality of life.

The analysis demonstrates that psychological comorbidity, specifically depression or anxiety, exerts the strongest influence on quality of life impairment among patients with rheumatoid arthritis. The effect size for this condition was the highest among all comorbidities examined, which suggests that emotional health plays a central role in shaping perceived disease burden. Cardiovascular disease followed closely, confirming its well-documented impact on morbidity and mortality in rheumatoid arthritis.

Osteoporosis and obesity also showed moderate to strong associations with reduced quality of life, likely reflecting their contribution to pain, reduced mobility and functional limitations. Diabetes mellitus exerted a moderate impact, consistent with its metabolic and systemic effects. Hypertension was associated with the weakest effect size although it remained clinically relevant given its high prevalence.

Overall, the ranking indicates that comorbidities do not influence rheumatoid arthritis outcomes uniformly. Instead, psychological and cardiovascular conditions emerge as dominant contributors to disease burden. These findings highlight the importance of targeted assessment and prioritized management of high-impact comorbidities in routine clinical care.

### 3.9. Differences Between Sexes and Age Groups

To explore whether the impact of comorbidities varies across demographic subgroups, the study assessed differences between sexes and across age categories. Previous research suggests that both sex related biological factors and age related disease accumulation may influence symptom severity, psychological burden and functional impairment. Table 13 presents the main variations observed in this sample, focusing on quality of life impact, emotional burden and functional limitations.

Clear demographic differences emerged regarding the perceived impact of comorbidities. Women reported significantly higher levels of quality of life impairment, emotional distress and functional limitations compared with men. These findings correspond with previous evidence suggesting that women with rheumatoid arthritis tend to experience more severe symptomatology, greater emotional burden and greater challenges in disease coping.

Marked variations were also observed across age groups. Participants younger than 40 years consistently reported the lowest burden across all examined domains including quality of life, emotional impact and daily functioning. The negative impact increased progressively with age, reaching its highest levels among participants older than 60 years. This pattern is consistent with the cumulative effect of longer disease duration combined with age related comorbidities.

Financial burden followed a similar trend. Older patients, especially those over 60 years, were more likely to report high financial strain compared with younger participants. This suggests that socioeconomic vulnerability intensifies with advanced age, possibly due to retirement, reduced income or increased healthcare needs.

Taken together, these findings indicate that both sex and age influence the perceived burden of comorbidities in rheumatoid arthritis. Women and older adults emerge as particularly vulnerable groups who may benefit from tailored clinical monitoring, psychological support and targeted social assistance.

### 3.10. Predictors of High Quality-of-Life Impairment

To identify independent predictors of high perceived quality-of-life impairment, a multivariable logistic regression model was performed. The dependent variable was high or very high QoL impact. Predictors included number of comorbidities, age, disease duration, sex, psychological comorbidity, and key somatic comorbidities. Odds ratios, confidence intervals and significance levels were calculated to determine the strength and direction of each association. To address the imbalance between comorbidity groups, multivariable logistic regression models were used, and effect estimates are presented with 95% confidence intervals to emphasize the precision and uncertainty of associations (Table 14).

The number of comorbidities emerged as one of the strongest predictors of high QoL impairment, with patients reporting three or more comorbidities showing an odds ratio of 4.62 (95% CI: 2.30–9.28). The width of the confidence interval reflects both the strength of the association and the variability inherent in the sample, highlighting the importance of interpreting point estimates together with their confidence intervals rather than relying solely on *p*-values.

### 3.11. Predictors of Difficulties in Disease Management

A second logistic regression model examined predictors of perceived difficulty in managing rheumatoid arthritis in the presence of comorbidities. The dependent variable was high or very high difficulty in disease management. Predictors mirrored those included in Model 1 to ensure comparability (Table 15).

Multimorbidity again demonstrated a strong predictive effect on perceived management difficulty, with patients with three or more comorbidities nearly four times more likely to report severe management challenges. Psychological distress was a robust predictor, tripling the odds of difficulty. Unlike Model 1, female sex reached statistical significance, indicating a differential experience in coping with the disease. Disease duration also contributed modestly. Obesity showed a small but significant effect, whereas cardiovascular disease and osteoporosis did not independently predict management difficulty.

The available literature highlights that comorbidities represent one of the most significant determinants of disease outcomes in patients with rheumatoid arthritis (RA), not only in terms of physical health, but also regarding psychological, social, and economic well-being [28]. Our study sought to investigate the prevalence of comorbidities among RA patients and to evaluate their perceived impact on quality of life, psychological health, daily activities, and access to care. The findings provide further evidence that comorbidities substantially exacerbate the burden of RA, confirming and extending observations from previous international reports [29,30].

Nearly all participants in our study reported at least one comorbidity, with the most common being hypertension, osteoporosis, cardiovascular disease, obesity, and depression/anxiety. These findings are consistent with those described in the literature, where cardiovascular and metabolic comorbidities are highly prevalent in RA and contribute significantly to both morbidity and mortality [5,29]. The strong correlation between the cumulative number of comorbidities and the reported deterioration in quality of life underscores the additive burden exerted by multiple chronic conditions. More than 80% of patients with two or more comorbidities perceived their health status as severely impaired, which aligns with studies indicating that multimorbidity amplifies functional decline and psychological distress [31,32].

The psychological impact of comorbidities was also noteworthy. Most patients reported that comorbidities negatively influenced their emotional state, with symptoms of depression and anxiety being common. These observations are in line with prior studies demonstrating a bidirectional relationship between comorbidity and psychological health: comorbidities exacerbate emotional distress, while poor psychological well-being worsens disease outcomes and treatment adherence [33]. Although only a minority of patients sought formal psychological support, many expressed a need for such interventions, suggesting that current healthcare services insufficiently address the psychosocial dimensions of RA.

Our findings also showed that comorbidities negatively influenced patients’ social relationships and professional life. The majority of participants reported difficulties in maintaining employment or daily activities due to the cumulative burden of RA and associated conditions. This observation resonates with prior studies showing that comorbidities in RA are strong predictors of disability, absenteeism, and work instability [34]. Interestingly, most participants felt partially or poorly understood by those around them, emphasizing the role of social stigma and lack of awareness as additional barriers to coping with the disease [35].

Access to healthcare services was generally rated positively in our cohort, with most patients reporting good access to medical consultations. However, patients also indicated that more frequent monitoring, better treatment availability, and enhanced educational support were essential for improving comorbidity management. These perceptions highlight a paradox observed in other studies: while access to routine care is acceptable, integrated, multidisciplinary, and patient-centred approaches remain insufficient [36].

Another important dimension explored in our study was the financial burden associated with managing RA and comorbidities. Most participants considered the costs to be high or very high, with medications, consultations, and investigations being the main drivers of expenses. This is consistent with previous findings that healthcare costs in RA are not only related to the disease itself but are largely driven by comorbidity management [37]. The majority of patients reported experiencing at least occasional financial difficulties, confirming that comorbidities substantially increase the economic burden of RA [38].

Support needs were strongly expressed by patients, with a clear emphasis on affordable treatments, frequent specialist consultations, structured rehabilitation, and psychological support. These findings mirror international literature that stresses the necessity of a holistic management strategy, addressing physical, psychological, and social needs simultaneously [39]. Educational programmes were also considered important by more than half of respondents, highlighting the demand for improved patient knowledge regarding multimorbidity and self-management [40].

Taken together, our results suggest that comorbidities in RA significantly deteriorate quality of life and generate a complex interplay of physical, psychological, social, and financial consequences. These findings align with the growing recognition that RA management cannot be limited to inflammation control alone. Instead, integrated care pathways: encompassing cardiovascular prevention, mental health support, rehabilitation, lifestyle interventions, and financial accessibility are essential for optimizing outcomes [41,42].

Future research should aim to further disentangle the causal relationships between comorbidities, psychological health, and socioeconomic outcomes, using longitudinal designs and validated instruments. Additionally, healthcare systems should prioritize integrated approaches, ensuring that RA patients benefit from coordinated multidisciplinary support tailored to their comorbidity profiles [43].

Our findings provide important insights into the multidimensional burden of comorbidities in patients with rheumatoid arthritis (RA), underlining both unmet needs and opportunities for improving integrated care. Despite advances in pharmacological treatment and the availability of effective disease-modifying therapies, many patients continue to experience substantial challenges in physical, emotional, social, and financial domains. This highlights the necessity of broadening the scope of RA management beyond inflammation control and joint protection, towards a more holistic model of care that actively addresses comorbidities and their consequences.

One of the most pressing unmet needs identified in this study is the requirement for enhanced medical monitoring. Patients frequently reported difficulties in managing RA in the presence of other chronic conditions, such as hypertension, cardiovascular disease, diabetes, and osteoporosis. Routine medical follow-up is essential not only for monitoring RA activity but also for early detection and treatment of associated comorbidities, which significantly contribute to morbidity and mortality. More structured and systematic monitoring protocols, integrated across specialties, would ensure that comorbidities are addressed in a timely and coordinated manner.

A second key finding relates to access to effective therapies. While biologic and targeted synthetic DMARDs have transformed the prognosis of RA, cost and availability remain significant barriers, especially in settings where healthcare resources are limited. Patients in our study frequently reported financial difficulties related to treatments and investigations, underscoring the need for policies that expand access to advanced therapies while ensuring affordability. The implementation of financial assistance programmes, together with the optimization of reimbursement schemes, could reduce treatment inequities and improve long-term disease control.

In addition, our results emphasize the importance of patient education as an integral component of care. A large proportion of patients expressed interest in better information regarding comorbidity management and disease self-care. Educational programmes tailored to RA patients could enhance adherence to therapy, empower patients to engage actively in disease management, and improve overall health literacy [44]. These programmes should cover both medical aspects (treatment regimens, monitoring requirements) and lifestyle factors (nutrition, physical activity, stress management), providing a comprehensive approach to daily disease coping strategies.

The psychological dimension represents another critical unmet need. Many participants reported emotional distress, anxiety, or depression linked to the presence of multiple comorbidities. Psychological support and counselling services, including structured interventions such as cognitive-behavioural therapy or support groups, should be integrated into the routine care pathway. These measures have the potential to reduce mental health burden, enhance resilience, and improve adherence to medical management, thereby contributing to better health outcomes.

Finally, the financial burden of managing RA and its comorbidities was evident in this study. Medication costs, consultations, investigations, and rehabilitation services were among the most frequently reported financial challenges. In line with these findings, there is a need for financial assistance programmes, such as subsidies for medications, coverage for multidisciplinary care, or targeted support for vulnerable groups. Addressing these barriers is critical to prevent treatment discontinuation and ensure equity in healthcare access.

Taken together, these findings suggest that optimizing patient outcomes in RA requires an integrated, multidisciplinary approach that goes beyond pharmacological therapy. Such an approach should combine regular medical monitoring, equitable access to effective treatments, comprehensive patient education, psychosocial support, and financial assistance programmes. By addressing these unmet needs, healthcare systems can move towards a model of care that not only controls disease activity but also significantly improves quality of life and reduces the overall burden of RA.

**Figure 1:**
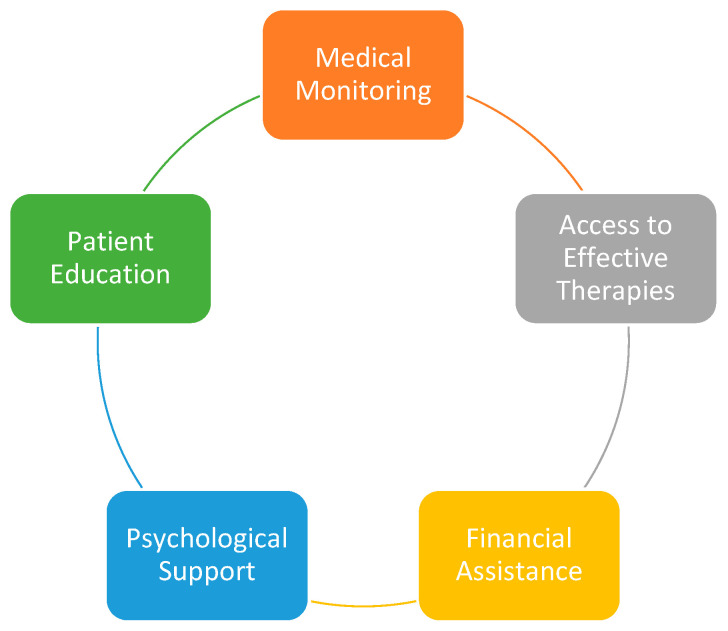
Conceptual framework for integrated care in RA with comorbidities.

The conceptual framework illustrates the multidimensional approach required to optimize patient outcomes and quality of life in rheumatoid arthritis (RA) complicated by comorbidities (Figure 1). At its centre lies the overarching goal of achieving improved health status and well-being, supported by five interconnected domains. Medical monitoring represents a fundamental pillar, emphasizing the importance of systematic follow-up, early detection of comorbid conditions, and coordinated interdisciplinary care. Access to effective therapies is equally critical, highlighting the role of biologics and targeted DMARDs, as well as the need to ensure affordability and reimbursement optimization to reduce treatment gaps. Patient education serves as another cornerstone, enabling individuals to better understand their disease, adopt healthier lifestyles through nutrition, exercise and stress management, and enhance adherence to therapeutic regimens. Psychological support is also essential, encompassing counselling, cognitive-behavioural therapy, group interventions and strategies to strengthen emotional resilience, particularly in the context of anxiety and depression. Finally, financial assistance underpins the sustainability of integrated care, addressing the burden of treatment costs through subsidies, reduction of out-of-pocket expenses and social protection programmes. Together, these domains provide a comprehensive model of integrated care designed to respond to unmet needs and promote holistic management of RA in the presence of comorbidities.

Collectively, the identification of high-risk phenotypes, the recognition of protective factors, the effect size hierarchy of comorbidities and the stratified demographic differences constitute an innovative analytical framework. This multidimensional approach moves beyond traditional descriptive reporting and provides clinically actionable insights that may support risk stratification, personalized management and improved resource allocation in rheumatoid arthritis care.

The multivariable logistic regression models further strengthen the evidence that the burden of comorbidities in rheumatoid arthritis extends beyond simple additive effects and reflects a complex interaction of somatic, psychological and demographic factors. In both models, the number of comorbidities emerged as one of the strongest independent predictors of adverse outcomes. Patients with three or more comorbidities displayed a fourfold increase in the likelihood of experiencing severe quality-of-life impairment and a similarly elevated risk of reporting substantial difficulties in disease management. These findings confirm that multimorbidity acts as a cumulative load that profoundly shapes patient experience, even after adjusting for age, disease duration and major somatic comorbidities.

Psychological distress, particularly depression or anxiety, demonstrated a remarkably strong and consistent effect across both models. The presence of psychological symptoms nearly quadrupled the odds of high-quality-of-life impairment and tripled the odds of difficulty in managing the disease. This reinforces the central role of mental health as a key determinant of outcomes in RA and suggests that routine psychological screening and early intervention should be integrated into standard care pathways.

The models also shed light on the nuanced contributions of demographic and clinical factors. Ageing and longer disease duration independently increased the likelihood of poorer outcomes, reflecting the cumulative nature of chronic disease. Female sex, although not a predictor of quality-of-life impairment in the adjusted model, significantly predicted higher difficulty in disease management, indicating potential sex-specific vulnerability in coping mechanisms or healthcare engagement. Among somatic comorbidities, cardiovascular disease contributed significantly to reduced quality of life, while obesity showed a small but significant effect on disease management difficulty. These findings suggest that the clinical significance of specific comorbidities may vary depending on the outcome evaluated.

Together, the logistic regression models provide important new insights beyond descriptive analyses by identifying independent predictors of patient burden. They clarify which characteristics place patients at greatest risk and highlight modifiable factors that may serve as intervention targets. The integration of multimorbidity burden, psychological vulnerability and demographic characteristics into predictive models advances the current understanding of risk stratification in RA and supports a move toward personalized, multidisciplinary management strategies designed to reduce disease impact and improve long-term outcomes.

## 4. Limitations of the Study

Limitations and generalizability. The present study relies primarily on self-reported data, including comorbidity status and perceived disease impact, which may introduce recall and reporting biases. Participants may under-report or over-report certain diagnoses and symptoms, and the study did not verify comorbidities through medical records, laboratory tests, or imaging. Consequently, prevalence estimates and effect sizes should be interpreted with caution, particularly for conditions that may be underdiagnosed or variably recognized by patients.

In addition, the mixed recruitment strategy (online dissemination via social media/WhatsApp/email and supervised completion among hospitalized patients) may have introduced selection bias. Online recruitment may disproportionately attract individuals who are more health-literate, more engaged in patient communities, or more motivated to report symptoms and unmet needs, while hospitalized participants may represent patients with more severe disease, higher comorbidity burden, or greater functional limitation. As a result, the sample may not fully represent the broader RA population, and the exceptionally high proportion of participants reporting at least one comorbidity (98.6%) may partly reflect these recruitment and self-selection mechanisms.

These sources of bias limit the external validity and generalizability of the findings. The results should therefore be considered hypothesis-generating and most applicable to patient groups similar to those captured by our recruitment channels (e.g., urban, digitally connected patients and those requiring hospital care). Future studies should aim to validate comorbidity status using clinical records, apply probability-based or registry sampling where feasible, and compare characteristics between online and hospital-recruited participants to quantify potential selection effects.

Another important limitation is the cross-sectional nature of the survey, which precludes any inference of causal relationships between comorbidities and quality of life, psychological distress, or social outcomes. The observed associations should therefore be interpreted strictly as correlational and reflect relationships at a single time point rather than directional or temporal effects. Longitudinal studies are needed to examine the progression of comorbidities over time and to clarify their long-term influence on disease management and health-related quality of life (HRQoL).

In addition, the study did not stratify participants according to treatment regimens, disease activity scores (e.g., DAS28), or the clinical severity of individual comorbidities, all of which may substantially influence patient-reported outcomes. The absence of these variables may have limited the ability to fully adjust for disease heterogeneity and treatment-related effects.

An additional limitation relates to the very small number of participants without comorbidities, which constrained the use of this subgroup as a reliable comparator in t-tests and regression analyses. Although statistically significant differences were observed, estimates derived from this comparison are inherently unstable and may be sensitive to small changes in sample composition. To mitigate this issue, the primary analytical focus was placed on comorbidity burden categories and on multivariable regression models with confidence intervals. Future studies should aim to include larger, well-balanced comparator groups to strengthen statistical inference.

The study did not perform a formal comparative analysis between online-recruited and hospital-recruited participants, which limits the ability to quantify the magnitude of these potential differences. Such analyses could provide valuable insights into how recruitment modality influences reported comorbidity burden, psychological distress, and perceived quality-of-life impairment. Future studies should consider stratifying analyses by recruitment source or applying weighting procedures to better approximate the characteristics of the broader RA population.

Given the cross-sectional design of the study, all observed relationships should be interpreted as associations rather than causal effects. Although the directionality of several associations identified in this study is biologically and clinically plausible and supported by prior longitudinal research, the present analysis captures correlations at a single time point and does not permit inference regarding temporal sequence or causality. To maintain methodological rigour, interpretations throughout the Discussion and Conclusions have been framed consistently in associative terms.

In terms of external validity, the findings should be interpreted primarily within the context of Romanian patients with rheumatoid arthritis who are actively engaged with healthcare services or patient networks. Differences in healthcare system organization, access to biologic therapies, socioeconomic conditions, and cultural perceptions of illness may limit the direct transferability of results to other regions or countries. Nevertheless, the identified associations between comorbidity burden and multidimensional patient-reported outcomes are likely relevant to other settings, even if prevalence estimates and effect sizes may vary.

## 5. Practical Implications

The findings of this study underscore the need for integrated, patient-centred care pathways that address not only rheumatoid arthritis disease activity but also the multidimensional burden associated with comorbidities. Although the proposed high-risk profiles are exploratory and require external validation, they provide a useful framework for conceptualizing unmet needs and structuring supportive care strategies within an integrated care model.

### 5.1. Patients with Severe Somatic Multimorbidity

Patients characterized by a high burden of somatic comorbidities (e.g., cardiovascular disease, diabetes, obesity, osteoporosis) reported substantial impairment in quality of life and functional capacity. For this subgroup, integrated medical monitoring may include:

- Coordinated follow-up across rheumatology, cardiology, endocrinology, and primary care, with clearly defined responsibilities and shared care plans;
- Regular cardiovascular and metabolic risk assessment, including blood pressure, lipid profiles, glycemic control, and fracture risk evaluation;
- Medication reconciliation and optimization to reduce polypharmacy risks and improve adherence.

Such approaches may help identify complications early and reduce fragmentation of care, particularly in patients with long disease duration and high healthcare utilization.

### 5.2. Patients with High Psychological Vulnerability

Patients reporting depression, anxiety, or high emotional impact experienced marked difficulties in disease management and reduced quality of life. For this subgroup, integrated psychological support may involve:

- Routine screening for depression and anxiety during rheumatology visits using brief, validated tools;
- Referral pathways to mental health professionals, including psychologists or psychiatrists, embedded within or closely linked to rheumatology services;
- Access to structured psychosocial interventions, such as cognitive–behavioural therapy, stress-management programmes, or peer support groups.

Addressing psychological vulnerability alongside somatic disease management may improve coping capacity, treatment adherence, and overall patient experience.

### 5.3. Patients with High Socioeconomic Burden

Participants experiencing significant financial strain and work-related limitations reported substantial barriers to effective disease management. For this subgroup, integrated care strategies may include:

- Assessment of financial burden and employment challenges as part of routine clinical encounters;
- Involvement of social workers or patient navigators to facilitate access to reimbursement schemes, disability support, or workplace accommodations;
- Simplified treatment pathways and follow-up schedules, aiming to reduce indirect costs associated with frequent visits or complex care regimens.

Such measures may help mitigate socioeconomic barriers that compound the clinical burden of multimorbidity.

### 5.4. Patient Education and Self-Management Support

Across all subgroups, patients expressed a strong need for improved education and information. Tailored educational interventions could include:

- Structured patient education programmes focusing on multimorbidity management, medication adherence, lifestyle modification, and symptom monitoring;
- Accessible educational materials adapted to different levels of health literacy and delivered through multiple formats (in-person, digital, written);
- Empowerment strategies that support shared decision-making and active patient participation in care planning.

### 5.5. Implications for Health Systems and Policy

From a health system perspective, the study highlights the potential value of multidisciplinary care models that integrate medical, psychological, and social support. While the present findings are exploratory, they suggest that risk-informed stratification approaches may help allocate resources more efficiently by identifying patients who could benefit most from intensified, coordinated support.

Future research should evaluate the feasibility, effectiveness, and cost-effectiveness of such integrated care pathways using longitudinal and interventional designs. Validation of high-risk profiles and testing of tailored interventions will be essential before clinical implementation.

## 6. Conclusions

These findings contribute novel evidence from an Eastern European cohort, a population that remains underrepresented in current research on comorbidities in rheumatoid arthritis.

This cross-sectional study indicates that comorbidity burden, psychological distress, and socioeconomic strain are strongly associated with poorer health-related quality-of-life outcomes and greater perceived difficulty in disease management among patients with rheumatoid arthritis. Beyond documenting the high prevalence of multimorbidity in this population, the study provides additional insights by exploring distinct, non-validated high-risk profiles characterized by combinations of somatic comorbidity burden, psychological vulnerability, and socioeconomic strain. These profiles were associated with worse patient-reported quality of life, reduced functional capacity, and increased management difficulties; however, they should be interpreted as exploratory and hypothesis-generating rather than clinically actionable phenotypes.

The ranking of comorbidities by effect size suggests that depression or anxiety and cardiovascular disease are most strongly associated with higher perceived disease burden, followed by osteoporosis, obesity, diabetes mellitus, and hypertension. These associations highlight domains that may warrant particular attention in future research and comprehensive patient assessment, rather than implying causal effects. At the same time, the identification of protective factors—such as younger age, shorter disease duration, higher perceived social support, and the absence of psychological distress—underscores the potential role of resilience-related factors in mitigating the perceived burden of multimorbidity.

Differences observed across sex and age groups further emphasize the heterogeneity of patient experience, with women and older adults reporting higher levels of quality-of-life impairment, emotional burden, and functional limitations. These demographic patterns point to variability in vulnerability rather than deterministic effects, reinforcing the importance of individualized assessment.

Overall, the study offers a multidimensional perspective on comorbidity burden in rheumatoid arthritis, illustrating the interplay between clinical, psychological, and socioeconomic factors as reported by patients. The findings support the need for further longitudinal and externally validated studies to clarify causal pathways, validate high-risk profiles, and assess the predictive value of identified associations. Such future work may inform the development of integrated and personalized care approaches aimed at improving patient-reported outcomes in rheumatoid arthritis.

Multivariable logistic regression analyses further indicated that higher comorbidity burden—particularly the presence of three or more comorbidities—and psychological distress were independently associated with poorer quality-of-life outcomes and greater perceived difficulty in disease management, after adjustment for demographic and clinical factors. Age, longer disease duration, and selected somatic comorbidities were also associated with adverse outcomes, while female sex was associated with greater management difficulty. Together, these results reinforce the multifactorial and associative nature of disease burden in rheumatoid arthritis and highlight the importance of comprehensive, multidisciplinary assessment rather than single-disease-focused evaluation.

Given the exploratory nature of the ICRA-Q questionnaire, the present findings should be viewed as indicative of associations rather than definitive estimates of health-related quality-of-life impairment. The study provides preliminary evidence regarding the multidimensional burden of comorbidities in rheumatoid arthritis and highlights domains that warrant further investigation using fully validated instruments and longitudinal designs.

## Figures and Tables

**Table 1:** Sociodemographic characteristics of study participants.

Variable	Category	N	%
Age (years)	<30	1	0.3
	30–40	18	6.3
	41–50	42	14.7
	51–60	116	40.6
	>60	109	38.1
Gender	Male	43	15.0
	Female	243	85.0
Residence	Urban	251	87.8
	Rural	35	12.2
Disease duration	<1 year	19	6.6
	1–5 years	97	33.9
	6–10 years	133	46.5
	>10 years	37	12.9

**Table 2:** Prevalence and impact of comorbidities among study participants.

Variable	Category	N	%
Other diagnosed conditions	Yes	282	98.6
	No	4	1.4
Type of comorbidities	Hypertension	202	70.9
	Osteoporosis	171	60.0
	Cardiovascular disease	166	58.2
	Obesity	114	40.0
	Diabetes mellitus	84	29.5
	Depression/Anxiety	63	22.1
Number of comorbidities affecting QoL	None	5	1.8
	1 comorbidity	52	20.4
	2–3 comorbidities	170	59.6
	>3 comorbidities	58	18.2
Perceived impact on health	Very much	63	22.0
	Much	194	67.8
	Moderate	63	22.0
	Little/None	12	4.2
Difficulty in RA management with comorbidities	Very difficult	81	28.3
	Difficult	195	68.2
	Moderate to none	10	3.5
Main difficulties reported	Increased pain	281	98.3
	Reduced mobility	273	95.5
	Fatigue	217	75.9
	Emotional problems	117	40.9
	Financial burden	66	23.1
Treatment for comorbidities	Yes	280	97.9
	No	6	2.1
Evaluation of access to healthcare	Good/Very good	273	92.3
	Satisfactory	13	4.5
	Poor/Very poor	0	0
Perceived medical support	Sufficient	246	86.0
	Insufficient	40	14.0
Suggested improvements	More effective treatments	275	96.2
	More frequent monitoring	268	93.7
	Better information/education	228	79.7
	Psychological support	167	58.4

**Table 3:** Psychological and social impact of comorbidities among study participants.

Variable	Category	N	%
Emotional impact of comorbidities	Very much	13	4.5
	Much	187	65.4
	Moderate	78	27.3
	Little	7	2.4
	None	1	0.4
Depression/Anxiety symptoms	Frequent	25	8.7
	Occasional	230	80.4
	Rare	31	10.8
	None	0	0.0
Psychological support (counselling)	Yes, frequent	18	6.3
	Yes, occasional	49	17.1
	Rarely	213	74.5
	None	6	2.1
Psychological support (support groups)	Yes, frequent	3	1.0
	Yes, occasional	49	17.1
	Rarely	118	41.3
	None	116	40.6
Psychological influence on RA management	Significant	28	9.8
	To some extent	216	75.5
	Slight	41	14.3
	None	1	0.4
Impact on relationships	Very much	10	3.5
	Much	209	73.1
	Moderate	57	19.9
	Little	9	3.1
	None	1	0.4
Difficulties with work/daily activities	Frequent	17	7.0
	Occasional	242	84.6
	Rare	20	7.0
	None	7	2.4
Perception of understanding by others	Complete	27	9.4
	Partial	254	88.8
	Low	5	1.8
	None	0	0.0

**Table 4:** Access to information, education, and financial burden among study participants.

Variable	Category	N	%
Understanding of comorbidities in RA	Very good	13	4.5
	Good	255	89.2
	Fair	18	6.3
	Poor/None	0	0.0
Sources of information	Rheumatologist	283	99.0
	Family physician	267	93.4
	Internet	131	45.8
	Patient associations	33	11.5
	Family/friends	28	9.8
Participation in educational programs	Definitely yes	134	16.1
	Yes, if free	105	36.7
	Maybe	46	46.9
	Not interested	1	0.3
Perceived financial burden	Very high	67	23.4
	High	199	69.6
	Moderate	17	5.9
	Low	3	1.1
	None	0	0.0
Main costs reported	Medications	275	96.2
	Diagnostic tests/imaging	247	86.4
	Medical consultations	244	85.3
	Physical therapy/rehabilitation	134	46.9
Financial difficulties due to disease	Frequent	30	10.5
	Occasional	228	79.7
	Rare	26	9.1
	None	2	0.7

**Table 5:** Support needs reported by study participants.

Variable	Category	N	%
Access to affordable treatments	Yes	275	96.2
More frequent consultations with specialists	Yes	273	95.5
Rehabilitation programs	Yes	254	88.8
Psychological support or peer groups	Yes	195	68.2
Expanded medical education	Yes	153	53.5

**Table 6:** Association between presence of comorbidities and perceived impact on quality of life (χ^2^ test).

Variable	Perceived QoL Impact (High/Very High)	Perceived QoL Impact (Moderate/Low)
No comorbidities (n = 5)	1 (20.0%)	4 (80.0%)
1 comorbidity (n = 52)	28 (53.8%)	24 (46.2%)
2–3 comorbidities (n = 170)	142 (83.5%)	28 (16.5%)
>3 comorbidities (n = 58)	53 (91.4%)	5 (8.6%)
Total (N = 285)	224 (78.6%)	61 (21.4%)

**Table 7:** Comparison of perceived quality of life scores between RA patients with and without comorbidities (independent samples *t*-test).

Group	Mean (M)	Standard Deviation (SD)	*t*	df	*p*-Value
With comorbidities (n = 282)	3.91	0.62	5.37	284	<0.001
Without comorbidities (n = 4)	2.25	0.50			

**Table 8:** One-way ANOVA of perceived general health impact by age group.

Age Group	n	Mean (M)	SD
<40 years (merged <30 & 30–40)	19	3.45	0.68
41–50 years	42	3.78	0.61
51–60 years	116	4.12	0.55
>60 years	109	4.20	0.50

**Table 9:** One-way ANOVA of perceived general health impact by disease duration.

Disease Duration	n	Mean (M)	SD
<1 year	19	3.50	0.65
1–5 years	97	3.79	0.62
6–10 years	133	4.08	0.57
>10 years	37	4.22	0.52

**Table 10:** High-risk patient subgroups based on comorbidity burden, psychological vulnerability and socioeconomic strain.

Subgroup	Definition (Criteria)	N (%)	Mean Quality of Life Impact Score	High Work or Daily Activity Difficulty (%)	High Financial Burden (%)
1. Severe Somatic Multimorbidity	At least three comorbidities including at least one major condition such as cardiovascular disease, diabetes or obesity	58 (20.3%)	4.35 ± 0.42	89.7%	56.9%
2. High Psychological Vulnerability	Presence of depression or anxiety, high emotional impact and psychological influence on rheumatoid arthritis management	63 (22.0%)	4.28 ± 0.47	82.5%	48.3%
3. High Socioeconomic Burden	High or very high financial burden, frequent financial difficulties and frequent work related limitations	72 (25.2%)	4.12 ± 0.51	94.1%	100%
4. Very High-risk Composite Phenotype	At least three comorbidities combined with depression or anxiety and high financial burden	34 (11.9%)	4.61 ± 0.39	97.1%	100%

**Table 11:** Protective factors associated with lower perceived disease burden and better quality of life in patients with rheumatoid arthritis.

Protective Factor	Definition	N (%)	Association with Lower QoL Impact	Statistical Significance (*p* Value)
Younger Age	Participants under 40 years of age	19 (6.6%)	Lower mean perceived impact score compared with older groups	<0.01
Shorter Disease Duration	Less than one year since diagnosis	19 (6.6%)	Reported fewer functional limitations and lower perceived burden	<0.01
Absence of Psychological Distress	No symptoms of depression or anxiety	31 (10.8%)	Significantly lower emotional impact and fewer difficulties managing RA	<0.001
Moderate Comorbidity Burden	Zero or one comorbidity	57 (22.2%)	Highest proportion of moderate or low QoL impact	<0.001
High Perceived Social Support	Participants reporting full or partial understanding from others	281 (98.2%)	Lower emotional strain and higher coping ability	<0.05
Good Access to Healthcare	Rating access as good or very good	273 (92.3%)	Reduced perceived difficulties in managing comorbidities	<0.05

**Table 12:** Ranking of comorbidities according to their effect size on quality of life impairment in rheumatoid arthritis.

Comorbidity	N (%)	Quality of Life Impact (High or Very High %)	Effect Size (Cramer’s V)	Strength of Impact
Depression or Anxiety	63 (22.1%)	91.0%	0.42	Strong
Cardiovascular Disease	166 (58.2%)	87.3%	0.36	Strong
Osteoporosis	171 (60.0%)	82.5%	0.28	Moderate to strong
Obesity	114 (40.0%)	79.8%	0.24	Moderate
Diabetes Mellitus	84 (29.5%)	76.1%	0.22	Moderate
Hypertension	202 (70.9%)	72.4%	0.18	Weak to moderate

**Table 13:** Differences between sexes and age groups in perceived health impact, emotional burden and functional limitations.

Variable	Male (n = 43)	Female (n = 243)	Younger than 40 Years (n = 19)	41 to 50 Years (n = 42)	51 to 60 Years (n = 116)	Older than 60 Years (n = 109)
High or Very High QoL Impact (%)	65.1%	80.2%	42.1%	68.3%	82.7%	88.1%
High Emotional Impact (%)	51.2%	73.8%	36.8%	58.5%	74.1%	79.8%
Frequent Difficulties with Work or Daily Activities (%)	69.8%	87.6%	47.4%	76.2%	88.8%	91.7%
High Financial Burden (%)	55.8%	72.0%	31.6%	58.5%	73.3%	79.8%

**Table 14:** Logistic regression model predicting high quality-of-life impairment (Model 1).

Predictor	OR	95% CI	*p*-Value
≥3 comorbidities vs. 0–1	4.62	2.30–9.28	<0.001
2–3 comorbidities vs. 0–1	3.11	1.72–5.63	<0.001
Age (per 10-year increase)	1.38	1.12–1.74	0.003
Disease duration >10 years	2.21	1.15–4.24	0.017
Female sex	1.54	0.86–2.75	0.142
Depression or anxiety	3.98	2.15–7.34	<0.001
Cardiovascular disease	1.82	1.12–2.98	0.015
Osteoporosis	1.47	0.91–2.39	0.108
Obesity	1.32	0.79–2.19	0.281

**Table 15:** Logistic regression model predicting high difficulty in RA management (Model 2).

Predictor	OR	95% CI	*p*-Value
≥3 comorbidities vs. 0–1	3.87	1.98–7.55	<0.001
2–3 comorbidities vs. 0–1	2.94	1.61–5.36	<0.001
Age (per 10-year increase)	1.19	0.98–1.47	0.078
Disease duration >10 years	1.72	1.01–2.96	0.046
Female sex	1.88	1.03–3.44	0.039
Depression or anxiety	3.12	1.72–5.65	<0.001
Cardiovascular disease	1.41	0.88–2.25	0.152
Osteoporosis	1.29	0.81–2.06	0.284
Obesity	1.67	1.01–2.76	0.046

## Data Availability

The data presented in this study are available on request from the corresponding author due to ethical and privacy restrictions, as the dataset contains sensitive patient-reported health information collected under institutional ethical approval.

## Abbreviations

ACR	American College of Rheumatology
ANOVA	Analysis of Variance
AS-WIS	Ankylosing Spondylitis Work Instability Scale
bDMARDs	Biologic Disease-Modifying Antirheumatic Drugs
CBT	Cognitive–Behavioural Therapy
COPD	Chronic Obstructive Pulmonary Disease
CRP	C-Reactive Protein
CVD	Cardiovascular Disease
DAS-28	Disease Activity Score-28 Joints
DMARDs	Disease-Modifying Antirheumatic Drugs
EGA	Evaluator Global Assessment
ESR	Erythrocyte Sedimentation Rate
GDPR	General Data Protection Regulation
GCP	Good Clinical Practice
HRQoL	Health-Related Quality of Life
ICH	International Council for Harmonisation of Technical Requirements for Pharmaceuticals for Human Use
ILD	Interstitial Lung Disease
IPAQ	International Physical Activity Questionnaire
LDA	Low Disease Activity
MFIS	Modified Fatigue Impact Scale
MI	Myocardial Infarction
NHIF	National Health Insurance Fund
NSAIDs	Non-Steroidal Anti-Inflammatory Drugs
NYHA	New York Heart Association
OR	Odds Ratio
PGA	Patient Global Assessment
PSQI	Pittsburgh Sleep Quality Index
QoL	Quality of Life
RA	Rheumatoid Arthritis
RWD	Real-World Data
SD	Standard Deviation
SDAI	Simplified Disease Activity Index
SMR	Standardised Mortality Ratio
tDMARDs/tsDMARDs	Targeted Synthetic Disease-Modifying Antirheumatic Drugs
TNF	Tumour Necrosis Factor
WHOQOL-BREF	World Health Organization Quality of Life Questionnaire, Brief Version

## Appendix A

**Questionnaire on the Impact of Comorbidities on Patients with Rheumatoid Arthritis (RA)—(ICRA-Q)**

**Dear Patient,**

Thank you for your willingness to take part in this study. The purpose of this questionnaire is to assess the impact of comorbidities on the lives of individuals living with rheumatoid arthritis (RA), in order to better understand the challenges faced and to identify potential solutions to improve quality of life.

**Purpose of the Study**

Through this questionnaire, we aim to collect information on:

- The prevalence of comorbidities associated with RA;
- How these conditions affect physical health, emotional wellbeing, and daily activities;
- Access to treatments and support needed to manage the illness.

**Your Participation**

Your responses are extremely valuable and will contribute to a better understanding of patients’ needs. Completing the questionnaire takes approximately 10–15 min. All answers will be kept confidential and used solely for research purposes. No personal data that could identify you directly will be requested.

**Instructions for Completion**

(1) Please read each question carefully.
(2) Select the answers that best describe your situation.
(3) If a question provides space for comments, please feel free to fill it in if you consider it necessary.

Participation in this questionnaire is voluntary, and you may choose to withdraw at any time, without any consequences.

**Section 1. Demographic Information**

**1. What is your age?**

- Under 30
- 30–40
- 41–50
- 51–60
- Over 60

**2. What is your gender?**

- Female
- Male

**3. Where do you live?**

- Urban area
- Rural area

**4. How long ago were you diagnosed with rheumatoid arthritis?**

- Less than 1 year
- 1–5 years
- 6–10 years
- Over 10 years

**Section 2. Information about Comorbidities**

**5. Do you have any other diagnosed medical conditions besides rheumatoid arthritis?**

- Yes
- No (If “No”, skip to Section 4)

**6. If yes, which of the following comorbidities have you been diagnosed with? (You may select more than one)**

- Diabetes
- High blood pressure
- Osteoporosis
- Cardiovascular disease
- Obesity
- Depression/Anxiety
- Other conditions (please specify): _______________

**7. How many of these comorbidities do you feel affect your quality of life?**

- None
- 1 comorbidity
- 2–3 comorbidities
- More than 3 comorbidities

**Section 3. Impact on Daily Life**

**8. To what extent do you feel these comorbidities affect your overall health status?**

- Very much
- A lot
- Moderately
- Slightly
- Not at all

**9. How difficult is it to manage RA in the presence of other health conditions?**

- Very difficult
- Difficult
- Moderately difficult
- Easy to manage
- No difficulty

**10. What are the main difficulties caused by the comorbidities? (You may select more than one)**

- Increased pain
- Reduced mobility
- Increased fatigue
- Emotional issues (anxiety, depression)
- Additional financial costs for treatments
- Other difficulties (please specify): _______________

**Section 4. Treatment and Support**

**11. Do you follow treatment for your comorbidities in addition to RA treatment?**

- Yes
- No

**12. How would you rate your access to the medical services required to manage your comorbidities?**

- Very good
- Good
- Satisfactory
- Unsatisfactory
- Very poor

**13. To what extent do you feel supported by healthcare professionals in managing your comorbidities?**

- To a very great extent
- To a great extent
- Neutral
- To a small extent
- To a very small extent

**14. What measures do you think would improve the management of comorbidities? (You may select more than one)**

- More frequent medical monitoring
- Access to more effective treatments
- Better information on managing multiple conditions
- Emotional or psychological support
- Other suggestions (please specify): _______________

**Section 5. Psychological and Emotional Impact**

**15. Do you feel that the presence of comorbidities affects your emotional state?**

- Very much
- A lot
- Moderately
- Slightly
- Not at all

**16. Have you experienced symptoms of depression or anxiety following your RA diagnosis or due to other comorbidities?**

- Yes, frequently
- Yes, occasionally
- Rarely
- Not at all

**17. Have you sought psychological or emotional support (e.g., counselling, support groups)?**

- Yes
- No, but I would like to
- No, I don’t feel it is necessary

**18. Do you believe your mental state affects how you manage your rheumatoid arthritis?**

- Yes, significantly
- Yes, to some extent
- Slightly
- Not at all

**Section 6. Social Impact and Personal Relationships**

**19. How do comorbidities affect your relationships with family, friends, or colleagues?**

- Very much
- A lot
- Moderately
- Slightly
- Not at all

**20. Have you had difficulty maintaining a job or performing daily activities due to your health conditions?**

- Yes, frequently
- Yes, occasionally
- Rarely
- Not at all

**21. Do you feel that those around you understand the challenges you face due to your illness and comorbidities?**

- Yes, completely
- Yes, partially
- Not really
- Not at all

**Section 7. Access to Information and Medical Education**

**22. How well do you understand the impact of comorbidities on rheumatoid arthritis?**

- Very well
- Well
- Satisfactorily
- Slightly
- Not at all

**23. Where do you obtain information about managing your illness and comorbidities? (You may select more than one)**

- Rheumatologist
- General practitioner
- Internet (medical websites, forums)
- Patient associations
- Family/Friends
- Other sources (please specify): _______________

**24. Would you participate in educational programmes about RA and comorbidities if they were available?**

- Yes, definitely
- Yes, if they are free
- Maybe, depending on the programme
- No, not interested

**Section 8. Costs and Financial Burden**

**25. Do you consider that managing RA and your comorbidities involves high financial costs?**

- Yes, very high
- High
- Moderate
- Low
- Not significant

**26. What are the main expenses related to managing your illness and comorbidities? (You may select more than one)**

- Medication
- Medical consultations
- Investigations (e.g., tests, imaging)
- Physiotherapy or rehabilitation
- Other costs (please specify): _______________

**27. Do you face financial difficulties due to necessary treatments or investigations?**

- Yes, frequently
- Yes, occasionally
- Rarely
- Not at all

**Section 9. Support**

**28. What kind of support do you believe would improve your quality of life? (You may select more than one)**

- Access to more affordable treatments
- More frequent consultations with specialists
- Physical rehabilitation programmes
- Psychological support or support groups
- More detailed medical education
- Other suggestions (please specify): _______________

**Thank you!**
